# The Association of Homocysteine and Diabetic Retinopathy in Homocysteine Cycle in Chinese Patients With Type 2 Diabetes

**DOI:** 10.3389/fendo.2022.883845

**Published:** 2022-06-29

**Authors:** Wei-Ming Luo, Zhi-Peng Zhang, Wei Zhang, Jing-Yang Su, Xiao-Qian Gao, Xu Liu, Wan-Ying Wang, Chang-Tao Jiang, Zhong-Ze Fang

**Affiliations:** ^1^ Department of Toxicology and Sanitary Chemistry, School of Public Health, Tianjin Medical University, Tianjin, China; ^2^ Department of Surgery, Peking University Third Hospital, Beijing, China; ^3^ Key Laboratory of Molecular Cardiovascular Science, Department of Physiology and Pathophysiology, School of Basic Medical Sciences, Ministry of Education, Peking University, Beijing, China; ^4^ Tianjin Key Laboratory of Environment, Nutrition and Public Health, Tianjin, China

**Keywords:** homocysteine, diabetic retinopathy, homocysteine cycle pathway, metabolism, type 2 diabetes

## Abstract

**Objective:**

This study aimed to explore the relationship between homocysteine (Hcy) and diabetic retinopathy (DR) and the impacts of the Hcy pathway on this relationship against this background.

**Methods:**

This study retrieved 1979 patients with type 2 diabetes (T2D) from the First Affiliated Hospital of Liaoning Medical University in Jinzhou, Liaoning Province, China. Multiple logistic regression was used to analyze the effects of Hcy cycle on the relationship between Hcy and DR. Spearman’s rank correlation analysis was used to analyze the correlation between risk factors related to DR progression and Hcy. Finally, the results of logistic regression were supplemented by mediation analysis.

**Results:**

We found there was a negative correlation between low concentration of Hcy and DR (OR : 0.83, 95%CI: 0.69-1). After stratifying all patients by cysteine (Cys) or Methionine (Met), this relationship remained significant only in low concentration of Cys (OR: 0.75, 95%CI: 0.61-0.94). Through the RCS curve, we found that the effect of Hcy on DR presents a U-shaped curve relationship. Mediating effect in Met and Hcy cycles was also significant [Total effect c (OR: 0.968, 95%CI: 0.938-0.998), Direct effect path c’ (OR: 0.969, 95%CI: 0.940-0.999), Path a (OR: 1.047, 95%CI: 1.004-1.091), Path b (OR: 0.964, 95%CI: 0.932-0.998)].

**Conclusions:**

The relationship between Hcy and DR presents a U-shaped curve and the homocysteine cycle pathway has an impact on it. And too low concentration of Hcy indicates a lack of other substances, such as vitamins. It is suggested that the progression of DR is the result of a combination of many risk factors. Further prospective studies are needed to determine the role of Hcy in the pathogenesis of DR.

## Introduction

Diabetes is a metabolic disorders illness caused by the absolute or relative lack of insulin secretion, and type 2 diabetes (T2D) is more common in patients. At present, the incidence of diabetes has risen sharply around the world and has become a global public health event. According to 10th Edition of International Diabetes Federation (IDF) in 2021 there were 537 million people aged 20-79 with diabetes worldwide. This number is expected to grow to 643 million by 2030, and it will rise to 783 million by 2045 (1). As a populous country with 110 million people living with diabetes, China has the largest number of diabetic patients in the world, posing a huge challenge to healthcare system in China (2). Diabetic retinopathy (DR), as a major microvascular complication, is one of the most dangerous complications of diabetes. The prevalence of DR among diabetic patients worldwide was 34.6%, and it can reach 40.3% in developed countries (3). There are 2.6 million people had been suffered from visual impairment due to DR until 2015 (4), and this number has been growing over time. In China, 40% of diabetic patients suffer from DR (5), and 1/10 of DR will develop into vision-threatening DR (6). So, DR is considered to be an important factor leading to visual impairment and even blindness in working-age diabetic patients.

Many studies have found that the development of DR is not solely due to hyperglycemia, many patients with well-controlled glycemia also developed DR (7–9). And there is no obvious symptoms in the early stage of DR (10), so most of the current effective treatments are aimed at the late stage of DR, which often accompanied by serious adverse reactions (11). Therefore, it is particularly important to discover early biomarkers of DR’ development and carry out intervention in time.

Homocysteine (Hcy) is a sulfur-containing amino acid formed from methionine (Met) metabolism. In the Hcy cycle, a part of Hcy generates Cys through the trans-sulfurization pathway, and the other part is remethylated to regenerate Met. Hyperhomocysteinemia (HHcy) has been widely recognized as a risk factor for cardiovascular disease. Although the specific mechanism is unclear, studies have shown that Hcy causes cardiovascular disease by damaging the vascular endothelium (12, 13). As a microvascular disease, DR is also affected by Hcy to a certain extent. The Hcy cycle can regulate the balance of Met and Cys, linking the folate cycle to promote methylation (14), and the interaction between the various substances in the cycle can also affect the effect of Hcy on DR. In recent years, more and more researches about the effect of Hcy on DR have been carried out. At present, some published literatures have provided evidence for the effect of various risk factors on DR (15, 16), but the specific effect mechanism has been still not clear so far. Therefore, it is very important to understand the risk factors of DR, which provides the evidence for reducing the risk of DR in T2D and develops monitoring and intervention in high-risk groups and early-stage patients to reduce the risk of blindness in diabetic patients.

In this study, we aimed to explore the relationship between Hcy and DR and the impacts of Met and Cys involved in the Hcy cycle pathway on this relationship.

## Materials and Methods

### Study Method and Population

**Figure 1:**
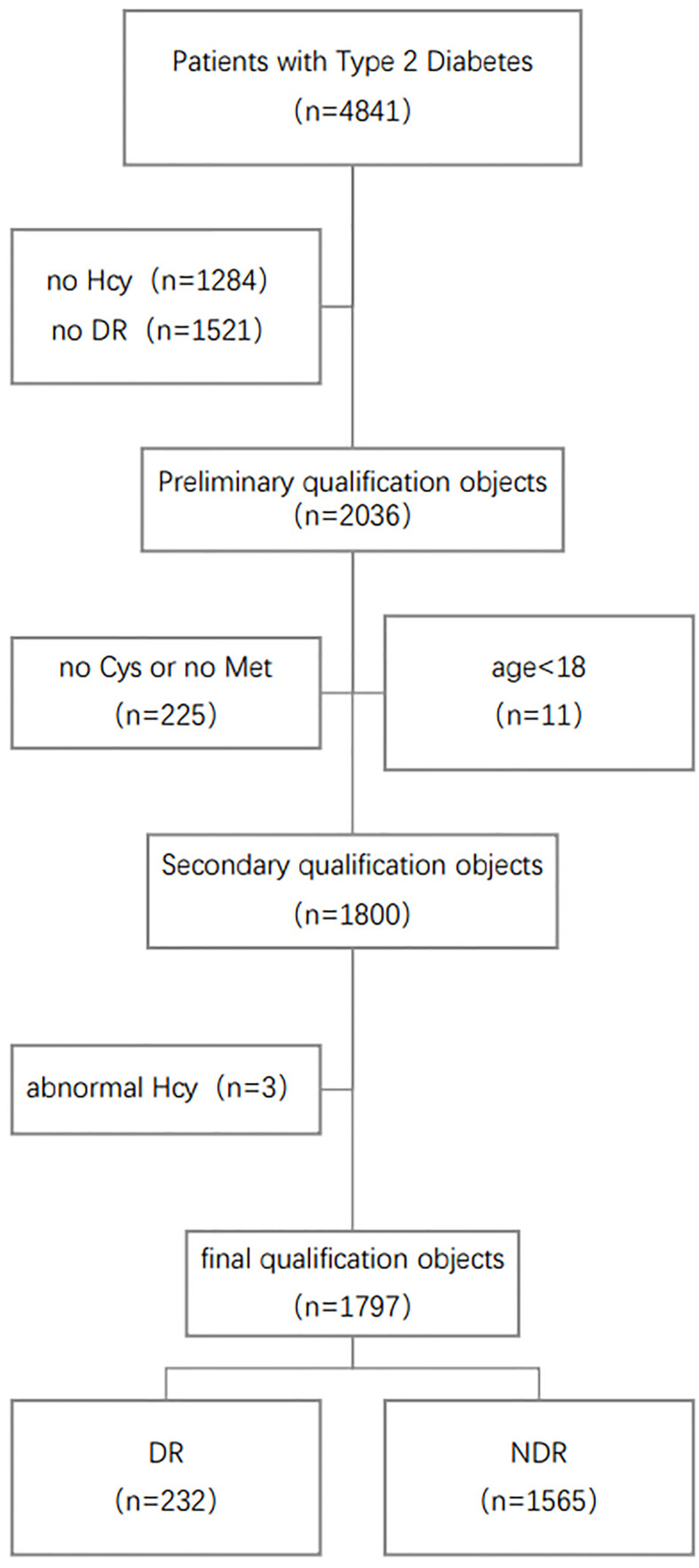
Schematic diagram of subject screening process. Hcy, Homocysteine; Met, Methionine; Cys, Cysteine; DR, Diabetic Retinopathy.

The First Affiliated Hospital of Liaoning Medical University (FAHLMU) is a tertiary general hospital located in Jinzhou, Liaoning Province, China. Inclusion criteria for this study were: 1) Patients diagnosed as T2D or treated with antihyperglycemic therapy; 2) Complete Hcy cycle and DR prevalence information. Exclusion criteria were: 1) T2D patients under 18 years old; 2) Subjects lacked the research indicators, height, weight, and blood pressure. Finally, a total of 1797 subjects were included in the present study, including 232 DR patients. The specific screening steps are shown in **Figure 1**. The diagnosis and classification of T2D in the present study were based on the standard published by World Health Organization (WHO) or treated with antihyperglycemic therapy (17). DR diagnostic criteria based on eye exam results for T2D (18).

The Ethics Committee for Clinical Research of FAHLMU approved the ethics of the study, and informed consent was waivered due to the retrospective nature of the study, which is consistent with the Declaration of Helsinki.

### Data Collection and Clinical Definitions

The data retrieved from the electronic medical records included demographic and anthropometric information, as well as current clinical factors and diabetic complications. Demographic included gender, age. Anthropometric measurements included height, weight, systolic blood pressure (SBP) and diastolic blood pressure (DBP). Clinical parameters included glycosylated hemoglobin (HbA1c), triglycerides (TG), total cholesterol (TC), high density lipoprotein cholesterol (HDL-C), low density lipoprotein cholesterol (LDL-C), urinary creatinine (UA), Serum creatinine (SCR), homocysteine (Hcy) ​​concentration, methionine (Met) concentration and cysteine (Cys)​​ concentration.

In the hospital, the measurements of anthropometric indicators were measured by using standardized procedures. Participants were allowed to wear light clothes and no shoes. Height and weight were measured to the nearest 0.5 cm and 0.1 kg, respectively. Blood pressure in adults was measured after a cuff on the right arm with a standard mercury sphygmomanometer and after a 10-minute rest in a seated position at an appropriate size. Age was obtained from the date of birth to the date of hospitalization or medical examination, and was calculated in years. The body mass index (BMI) was calculated as the ratio of body weight (kg) to squared height (m) and classified according to the overweight and obesity criteria recommended by the National Health Commission of China (19). DR was assessed by bilateral retinal photographs and defined as present if the following lesions were found: microaneurysm, retinal hemorrhage, soft exudate, hard exudate, or vitreous hemorrhage.

Based on the RCS curve, we defined the population with Cys concentration higher than 1.45 μmol/L as high Cys population, and the population with less than 1.45 μmol/L concentration as low Cys population (**Figure S1**); The population with higher than 16.9 μmol/L Met concentration was defined as the high Met population, and the population lower than 16.9 μmol/L was defined as the low Met population (**Figure S2**).

### Laboratory Assay

Dried blood spots were used in the metabolomic assay, which were prepared from capillary whole blood through an 8-h fasting. We measured the metabolites by direct infusion mass spectrometry technology equipped with the AB Sciex 4000 QTrap system (AB Sciex, Framingham, MA, USA). High-purity water (7732–18–5) and acetonitrile (75-05-8) were purchased from Thermo Fisher (Waltham, MA, USA), and were utilized as diluting agent and mobile phase. 1-Butanol(71-36-3) and acetyl chloride(75-36-5) were obtained from Sigma-Aldrich (St Louis, MO, USA). Isotope-labeled internal standard samples of amino acids (NSK-A) were purchased from Cambridge Isotope Labo-ratories (Tewksbury, MA, USA), while standard samples of the Hcy(14857-77-3), Cys(52-90-4) and Met(63-68-3) were purchased from Chrom Systems (Grafelfing, Germany). In brief, 8.5 mL of venous blood was drawn from each participant at 08:00 to 09.30 h in the morning after an 8-h fasting. Laboratory tests were carried out at a specialized diagnostic laboratory. The level of lipid profiles was analyzed with an automatic biochemistry analyzer (Hitachi 7150, Tokyo, Japan). We also assayed the level of HDL-C and LDL-C by the selective solubilization.

### Statistical Analysis

Continuous data were expressed as mean ± standard deviation (SD), non-normal distributed data were expressed as median (interquartile range), and categorical variables were expressed as numbers (percentages). In the four large groups divided by Cys and Met concentrations, it was tested whether there were differences between the patients in the DR group and the NDR group. The p-values of the repeated groups were FDR corrected. Continuous and normal distributed variables were tested with t-test or ANOVA, non-normal variables were analyzed by nonparametric test, and categorical variables were analyzed by chi-square test.

Binary logistic regression models were used to obtain odds ratios (OR) and the 95% confidence intervals (95%CI). Traditional risk factors for DR in T2D patients were adjusted through a structural adjustment program. Through the analysis, we obtained the unadjusted OR value and the adjusted OR value after adding traditional risk factors, including age, gender, body mass index, systolic blood pressure, diastolic blood pressure, low-density lipoprotein cholesterol, High-density lipoprotein cholesterol, triglyceride, total cholesterol, glycosylated hemoglobin, uric acid, serum creatinine.

Restrictive cubic spline (RCS) is a curve that can provide a more intuitive relationship. According to the change of the RCS curve, we select suitable nodes in the RCS. We used it to obtain cut-off values for metabolites associated with DR risk, and selected a cut-off point by visually inspecting the curves of DR probability. Then we repeated the logistic regression analysis in the Cys and Met populations to obtain the corresponding OR values to check whether the concentrations of Met or Cys would affect the relationship between Hcy and DR. Spearman correlation analysis confirmed the correlation between various factors in the Hcy cycle, Hcy and traditional related variables, and then through the mediation effect analysis, we analysis whether there is a mediating effect between Hcy cycle pathway and DR. We excluded patients with DR for >2 years to examine the effect of Hcy on the risk of DR. All analyses were performed using R version 4.1.0 and SAS 9.4.

## Result

### Description of Study Subjects

**Table 1** summarizes the selected characteristics of the DR group and the control group in the total population and the population grouped by Cys and Met. The study included a total of 1797 participants with a mean age of 57.27 years (SD: 14.17) and a mean BMI of 26.14 kg/m² (SD: 4.66). 48.1% of subjects were male. 232 DR patients were included in the study, of which 119 (51.3%) were male. The power of all four groups was greater than 0.95.

**Table 1:** Clinical and biochemical characteristics of participants according to the occurrence of diabetic retinopathy.

Variables	Total subjects	Low Cys		P	High Cys		P	Low Met		P	High Met		P
		Non-DR	DR		Non-DR	DR		Non-DR	DR		Non-DR	DR	
		Mean/number (SD or %)	Mean/number (SD or %)		Mean/number (SD or %)	Mean/number (SD or %)		Mean/number (SD or %)	Mean/number (SD or %)		Mean/number (SD or %)	Mean/number (SD or %)	
Age (years)	57.27 ± 14.17	56.66 ± 14.72	58.64 ± 11.05	0.158	57.67 ± 14.42	56.99 ± 11.28	0.632	57.11 ± 14.62	58.43 ± 10.66	0.341	57.24 ± 14.53	57.21 ± 11.71	0.981
Male sex	864 (48.1%)	361 (47.4%)	62 (51.7%)	0.445	384 (47.8%)	57 (50.9%)	0.603	348 (48.4%)	60 (49.6%)	0.886	397 (46.9%)	59 (53.2%)	0.257
Weight (kg)	71.73 ± 14.03	72.15 ± 14.49	70.58 ± 12.81	0.263	71.36 ± 14.52	69.53 ± 12.95	0.205	72.49 ± 14.63	70.69 ± 13.29	0.206	70.84 ± 13.65	69.36 ± 13.21	0.279
Height (cm)	165.00 (160.00, 172.00)	165.00 (160.00, 172.00)	164.00 (160.00, 171.00)	0.490	167.00 (160.00, 172.00)	165.00 (160.00, 170.00)	0.080	165.00 (160.00, 171.50)	163.00 (160.00, 172.00)	0.302	165.00 (160.00, 171.00)	164.00 (160.00, 171.00)	0.646
BMI (kg/m²)	26.14 ± 4.66	26.34 ± 5.18	25.72 ± 3.88	0.215	25.64 ± 4.63	25.33 ± 4.26	0.498	26.52 ± 5.25	26.01 ± 4.75	0.320	25.71 ± 4.31	25.18 ± 3.85	0.219
BMI<18.5	57 (3.2%)	20 (2.6%)	1 (0.9%)		37 (4.6%)	5 (4.5%)		26 (3.6%)	2 (1.7%)		33 (3.9%)	3 (2.7%)	
BMI≥18.5and<24.0	534 (29.7%)	235 (30.9%)	42 (35.0%)		251 (31.2%)	37 (33.0%)		209 (29.1%)	39 (32.2%)		254 (30.0%)	44 (39.6%)	
BMI≥24and<28.0	655 (36.4%)	275 (36.1%)	46 (38.3%)		308 (38.3%)	45 (40.2%)		243 (33.8%)	50 (41.3%)		334 (39.5%)	38 (34.2%)	
BMI≥28.0	551 ((30.7%)	231 (30.4%)	31 (25.8%)		208 (25.9%)	25 (22.3%)		241 (33.5%)	30 (24.8%)		225 (26.6%)	26 (23.5%)	
SBP (mmHg)	140.96 ± 23.92	140.55 ± 24.02	145.85 ± 25.09	0.026	140.14 ± 23.30	144.20 ± 24.58	0.087	139.04 ± 23.93	143.99 ± 24.37	0.036	141.42 ± 23.38	145.95 ± 25.21	0.058
DBP (mmHg)	83.24 ± 13.69	83.35 ± 13.18	83.91 ± 14.70	0.669	82.88 ± 13.89	84.25 ± 14.11	0.328	82.87 ± 14.29	83.78 ± 14.39	0.519	83.27 ± 12.93	84.17 ± 14.47	0.497
HbA1c (%)	9.62 ± 2.42	9.81 ± 2.47	9.31 ± 2.33	0.041	9.32 ± 2.36	9.84 ± 2.56	0.032	9.62 ± 2.37	9.74 ± 2.60	0.613	9.44 ± 2.36	9.37 ± 2.38	0.750
Triglyceride (mmol/L)	1.66 (1.12, 2.52)	1.69 (1.11, 2.54)	1.80 (1.21, 2.75)	0.267	1.67 (1.13, 2.55)	1.64 (1.17, 2.82)	0.441	1.71 (1.13, 2.59)	1.58 (1.11, 2.38)	0.376	1.62 (1.07, 2.43)	1.91 (1.33, 3.00)	0.005
TC (mmol/L)	4.76 (4.01, 5.54)	4.69 (4.01, 5.40)	4.80 (4.16, 5.74)	0.206	4.73 (3.95, 5.52)	5.11 (4.38, 5.98)	0.001	4.80 (3.98, 5.49)	4.96 (4.18, 5.74)	0.097	4.69 (3.93, 5.42)	4.88 (4.16, 5.65)	0.071
HDL-C (mmol/L)	1.11 ± 0.35	1.07 ± 0.32	1.12 ± 0.37	0.097	1.14 ± 0.38	1.15 ± 0.42	0.732	1.09 ± 0.34	1.16 ± 0.41	0.038	1.11 ± 0.36	1.11 ± 0.30	0.946
Male (HDL-C<1.0 mmol/L)	330 (18.4%)	161 (21.2%)	27 (22.5%)		131 (16.3%)	20 (17.9%)		138 (19.2%)	17 (14.1%)		147 (17.4%)	20 (18.0%)	
Female (HDL-C<1.3 mmol/L)	726 (40.4%)	330 (43.4%)	43 (35.8%)		313 (38.9%)	43 (38.4%)		296 (41.2%)	48 (39.7%)		347 (41.0%)	44 (39.6%)	
LDL-C (mmol/L)	2.83 (2.25, 3.46)	2.80 (2.28, 3.45)	2.80 (2.26, 3.50)	0.948	2.82 (2.24, 3.42)	3.13 (2.57, 3.51)	0.004	2.91 (2.32, 3.45)	2.81 (2.32, 3.40)	0.665	2.73 (2.22, 3.42)	2.89 (2.29, 3.46)	0.259
Hcy (μmol/L)	8.18 (7.47, 8.84)	7.98 (6.63, 8.55)	7.67 (6.46, 8.30)	0.007	8.41 (7.87, 9.19)	8.58 (7.88, 9.10)	0.507	8.16 (7.41, 8.71)	7.93 (7.05, 8.63)	0.109	8.24 (7.60, 8.97)	8.22 (6.95, 9.03)	0.656
<7.7μmol/L	536 (29.8%)	300 (39.4%)	62 (51.7%)		152 (18.9%)	22 (19.6%)		225 (31.3%)	46 (38.0%)		227 (26.8%)	38 (34.2%)	
≥7.7μmol/L	1261 (70.2%)	461 (60.6%)	58 (48.3%)		652 (81.1%)	90 (80.4%)		494 (68.7%)	75 (62.0%)		619 (73.2%)	73 (65.8%)	
UA	278.00 (181.00, 364.00)	284.20 (214.96, 371.00)	305.00 (238.25, 375.02)	0.149	265.70 (7.00, 358.00)	284.05 (175.03, 358.50)	0.460	282.00 (165.50, 369.50)	289.00 (196.00, 371.00)	0.779	273.55 (171.25, 360.00)	291.96 (228.50, 353.50)	0.178
SCR	59.00 (47.90, 75.51)	58.80 (47.52, 74.45)	56.12 (47.48, 75.26)	0.902	59.85 (47.03, 77.00)	59.52 (50.97, 74.22)	0.813	58.00 (46.05, 75.40)	56.74 (47.61, 74.89)	0.803	60.60 (49.16, 75.96)	57.00 (48.89, 72.43)	0.326

BMI, body mass index; SBP, systolic blood pressure; DBP, diastolic blood pressure; HbA1c, glycated hemoglobin; TG, triglyceride; HDL-C, high-density lipoprotein cholesterol; LDL-C, low-density lipoprotein cholesterol; Hcy, Homocysteine; UA, uric acid; SCR, serum creatinine.

Data are mean ± standard deviation, median (IQR), or n (%).

P values were derived from the t-test for normally distributed variables, Mann-Whitney U test for skewed distributions, Chi-square test (or fisher test if appropriate) for categorical variables. P < 0.05 was defined as statistically significant.

According to the Cys concentration, the population was divided into two groups. In the low Cys population, Only the differences of SBP, HbA1c and Hcy between two groups were statistically significant. DR patients had higher SBP, HbA1c concentration and lower Hcy concentration. In the high Cys population, the differences of HbA1c, TC and LDL-C between two groups among other indicators were statistically significant. The concentration of HbA1c, TC and HDL-C in the DR group were all higher.

We divided the general population into two groups according to the Met concentration of subjects in the same way. In the low Met population, the differences of SBP and HDL-C between two groups were statistically significant. The SBP and HDL-C concentrations in the DR group were higher. In the high Met population, except for TG, there was no significant difference in other indicators between the two groups, and the TG concentration was higher in the DR group.

### The Association Between Homocysteine and Diabetic Retinopathy

**Figure 2:**
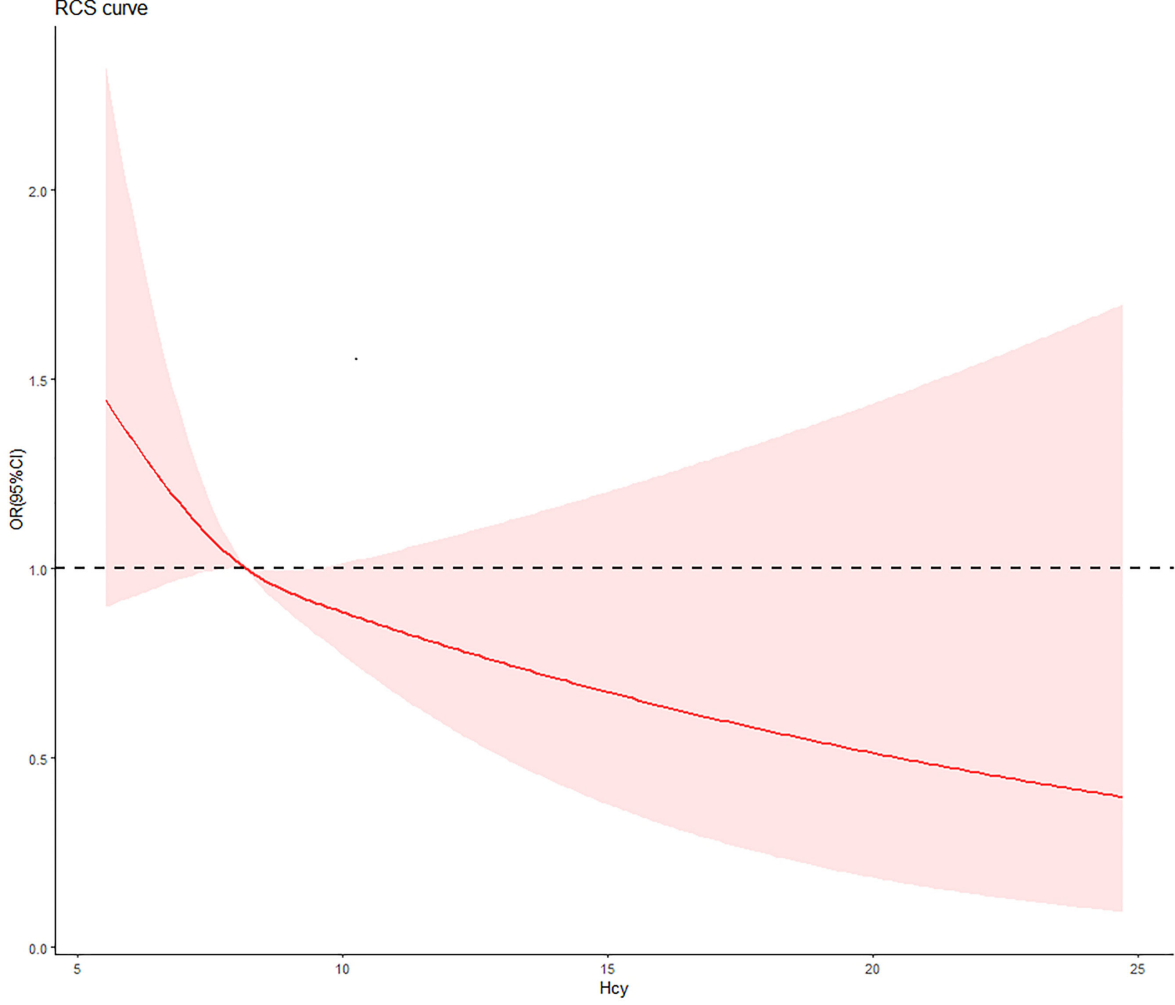
The relationship between Hcy concentration and DR risk in T2D patients. The red curve was derived from multivariate analysis that adjusted for age, gender, body mass index, systolic blood pressure, diastolic blood pressure, low-density lipoprotein cholesterol, high-density lipoprotein cholesterol, triglyceride, glycosylated hemoglobin, urinary creatinine and serum creatine.

The slope of the RCS curves of Hcy and DR have a process from large to small, and 7.7μmol/L is the junction point of the slope change. Among all subjects, 536 (29.8%) had plasma Hcy levels below 7.7 μmol/L (**Figure 2**).

For numerical Hcy, Hcy was inversely associated with DR risk in univariate regression, but the difference in this relationship was not significant (OR: 0.85, 95%CI: 0.71-1.03). After adjusting for traditional risk factors associated with DR, the negative correlation became significant and enhanced. After a step-by-step adjustment of the Model 1, we found that it was the adjustment of SBP that made the relationship between Hcy and DR significant. For the categorical Hcy (as reference), the relationship between Hcy and DR was significant in both univariate and multivariate regression (**Table 2**).

**Table 2:** Odds ratio of Homocysteine (Hcy) for the risk of diabetic retinopathy.

	OR(95%CI)	P
Univariable model		
Hcy, per μmol/L	0.85 (0.71,1.03)	0.064
<7.7μmol/L	reference	
≥7.7μmol/L	0.72 (0.54-0.96)	0.025
Multivariable model1		
Hcy, per μmol/L	0.84 (0.7,1)	0.027
<7.7μmol/L	reference	
≥7.7μmol/L	0.73 (0.55,0.98)	0.04
Multivariable model2		
Hcy, per μmol/L	0.83 (0.69,1)	0.024
<7.7μmol/L	reference	
≥7.7μmol/L	0.72 (0.53-0.96)	0.029
Multivariable model3		
Hcy, per μmol/L	0.83 (0.69,1)	0.025
<7.7μmol/L	reference	
≥7.7μmol/L	0.73 (0.54-0.98)	0.04

In different models, the categorical Hcy divided into two groups by 7.7μmol/L was used as a reference, and then observed changes of numerical and categorical Hcy respectively.

Multivariable Model 1 was adjusted for age, gender, body mass index., systolic blood pressure, diastolic blood pressure.

Multivariable Model 2 was adjusted for variables in Model 1 and concentrations of low-density lipoprotein cholesterol, high-density lipoprotein cholesterol, triglyceride, total cholesterol, glycosylated hemoglobin.

Multivariable Model 3 was adjusted for variables in Model 2 and concentrations of uric acid, serum creatinine.

### The Influence of Methionine and Cysteine Levels on the Association Between Homocysteine and Diabetic Retinopathy

In the population grouped by Cys concentration, the relationship between Hcy and DR was only significant in the low Cys population. Both univariate regression (OR: 0.77, 95%CI: 0.63-0.93) and multivariate regression (OR: 0.75, 95%CI: 0.61-0.94) showed a negative relationship between numerical Hcy and DR. For the binary Hcy, this negative relationship was still significant. This negative association enhanced after adjusting for traditional risk factors (univariate regression (OR: 0.61, 95%CI: 0.41-0.9), multivariate regression (OR: 0.6, 95%CI: 0.4-0.9). In the high Cys population, the relationship between Hcy and DR was no longer significant in both univariate and multivariate regression.

In addition, after grouping the total population by Met level, the relationship between Hcy and DR became no longer significant. The mediating effect of Hcy and Met cycle on DR was significant. It was suggested that the grouping of Met concealed the heterogeneity of Hcy levels in the same pathway (**Table 3**).

**Table 3:** Odds ratio of Homocysteine (Hcy) for the risk of diabetic retinopathy in different group by Met and Cys.

	Low Cys		High Cys		Low Met		High Met	
	OR (95%CI)	P	OR (95%CI)	P	OR (95%CI)	P	OR (95%CI)	P
Univariable model									
Hcy, per μmol/L	0.77 (0.63,0.93)	0.016	0.94 (0.75,1.17)	0.545	0.88 (0.69,1.13)	0.271	0.84 (0.64,1.11)	0.166	
<7.7μmol/L	reference		reference		reference		reference		
≥7.7μmol/L	0.61 (0.41,0.9)	0.024	0.95 (0.58,1.57)	0.853	0.74 (0.5,1.11)	0.148	0.7 (0.46,1.07)	0.108	
Multivariable model1									
Hcy, per μmol/L	0.76 (0.62,0.93)	0.016	0.91 (0.72,1.14)	0.374	0.87 (0.68,1.11)	0.208	0.83 (0.63,1.1)	0.152	
<7.7μmol/L	reference		reference		reference		reference		
≥7.7μmol/L	0.6 (0.4,0.89)	0.022	0.96(0.58,1.58)	0.873	0.74 (0.5,1.12)	0.158	0.71 (0.46,1.08)	0.117	
Multivariable model2									
Hcy, per μmol/L	0.76 (0.61,0.93)	0.016	0.93 (0.74,1.16)	0.498	0.87 (0.68,1.12)	0.239	0.84 (0.64,1.1)	0.162	
<7.7μmol/L	reference		reference		reference		reference		
≥7.7μmol/L	0.59 (0.4,0.89)	0.022	1.01 (0.61,1.68)	0.938	0.73 (0.48,1.1)	0.138	0.71 (0.46,1.09)	0.12	
Multivariable model3									
Hcy, per μmol/L	0.75 (0.61,0.94)	0.022	0.93 (0.74,1.16)	0.502	0.86 (0.66,1.12)	0.216	0.86 (0.66,1.13)	0.225	
<7.7μmol/L	reference		reference		reference		reference		
≥7.7μmol/L	0.6 (0.4,0.9)	0.030	1.01 (0.61,1.69)	0.940	0.72 (0.47,1.1)	0.131	0.74 (0.48,1.14)	0.175	

In different models, the categorical Hcy divided into two groups by 7.7μmol/L was used as reference, and then observed changes of numerical and categorical Hcy respectively.

Multivariable Model 1 was adjusted for age, gender, body mass index., systolic blood pressure, diastolic blood pressure.

Multivariable Model 2 was adjusted for variables in Model 1 and concentrations of low-density lipoprotein cholesterol, high-density lipoprotein cholesterol, triglyceride, total cholesterol, glycosylated hemoglobin.

Multivariable Model 3 was adjusted for variables in Model 2 and concentrations of uric acid, serum creatinine.

The p-values of the repeated groups were FDR corrected.

### Correlation Between Homocysteine Cycle and Relevant Factors

**Figure 3:**
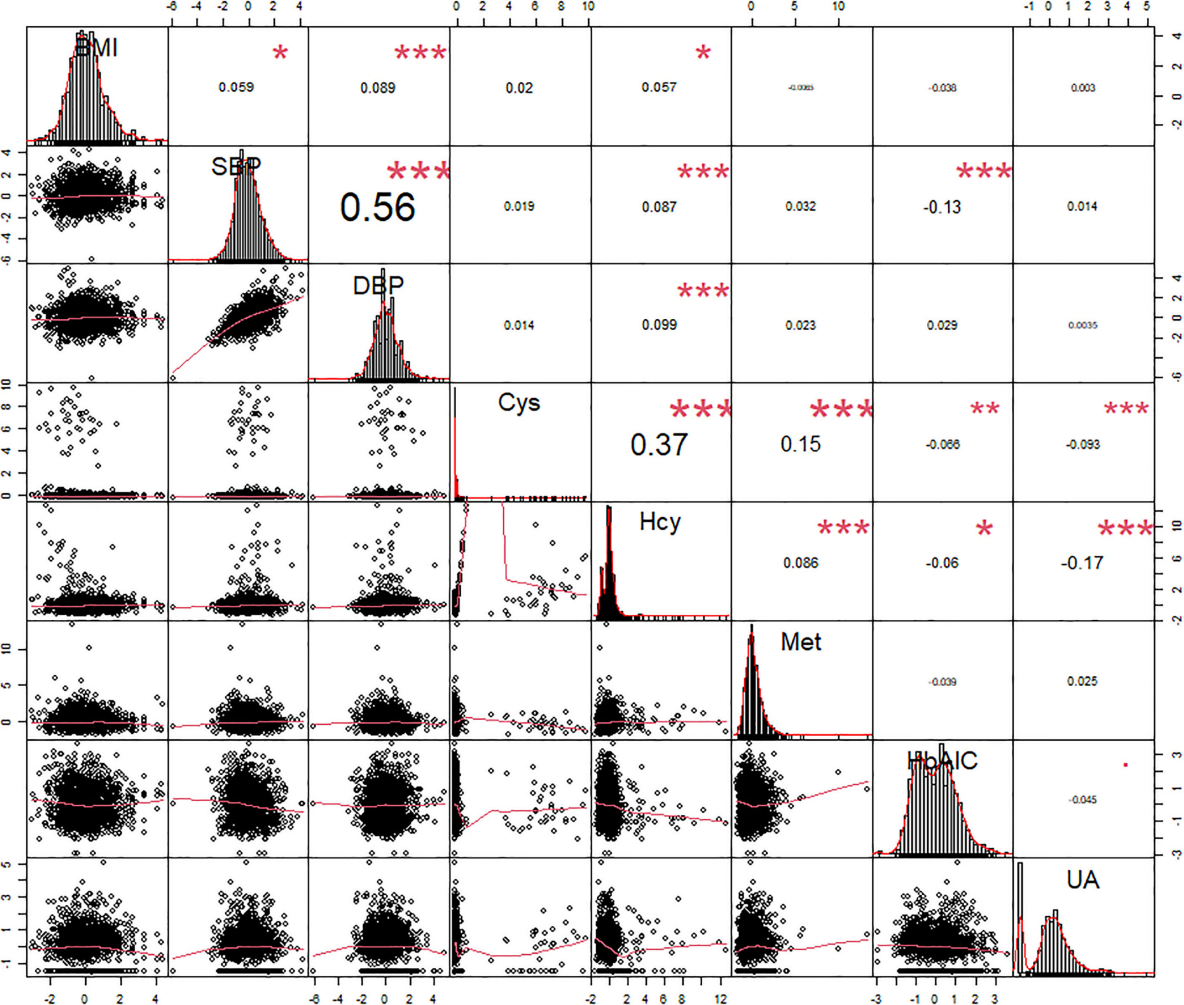
Spearman correlation coefficients and probability density plots between plasma Hcy and risk factors of DR (n=1797). BMI, body mass index. SBP, systolic blood pressure. DBP, diastolic blood pressure; HbAlc, glycated hemoglobin; Hcy, Homocysteine; Met, Methionine; Cys, Cysteine; UA, uric acid. *p-value <0.05, **p-value <0.01, ***p-value <0.001.

We analyzed the correlation between three main factors involved in Hcy cycle pathway and other factors associated with the progression of DR, and only retained the factors that were significantly associated with Hcy (**Figure 3**). At first, the positive correlations between Hcy, Met and Cys were all significant, and the correlation between Hcy and Cys was the largest, r=0.37. In addition, there were significant correlations between Hcy and BMI, SBP, DBP, HbA1c, and UA, among which HbA1c and UA were negatively correlated. In addition to the Hcy-related indicators, the factor with the strongest correlation with Hcy was UA, r=-0.17. The small correlation coefficient may be affected by other unadjusted factors, but the significance of its correlation indicates the significance of our inclusion of adjustment.

### Mediating Effect of Homocysteine Cycle Pathway

**Figure 4:**
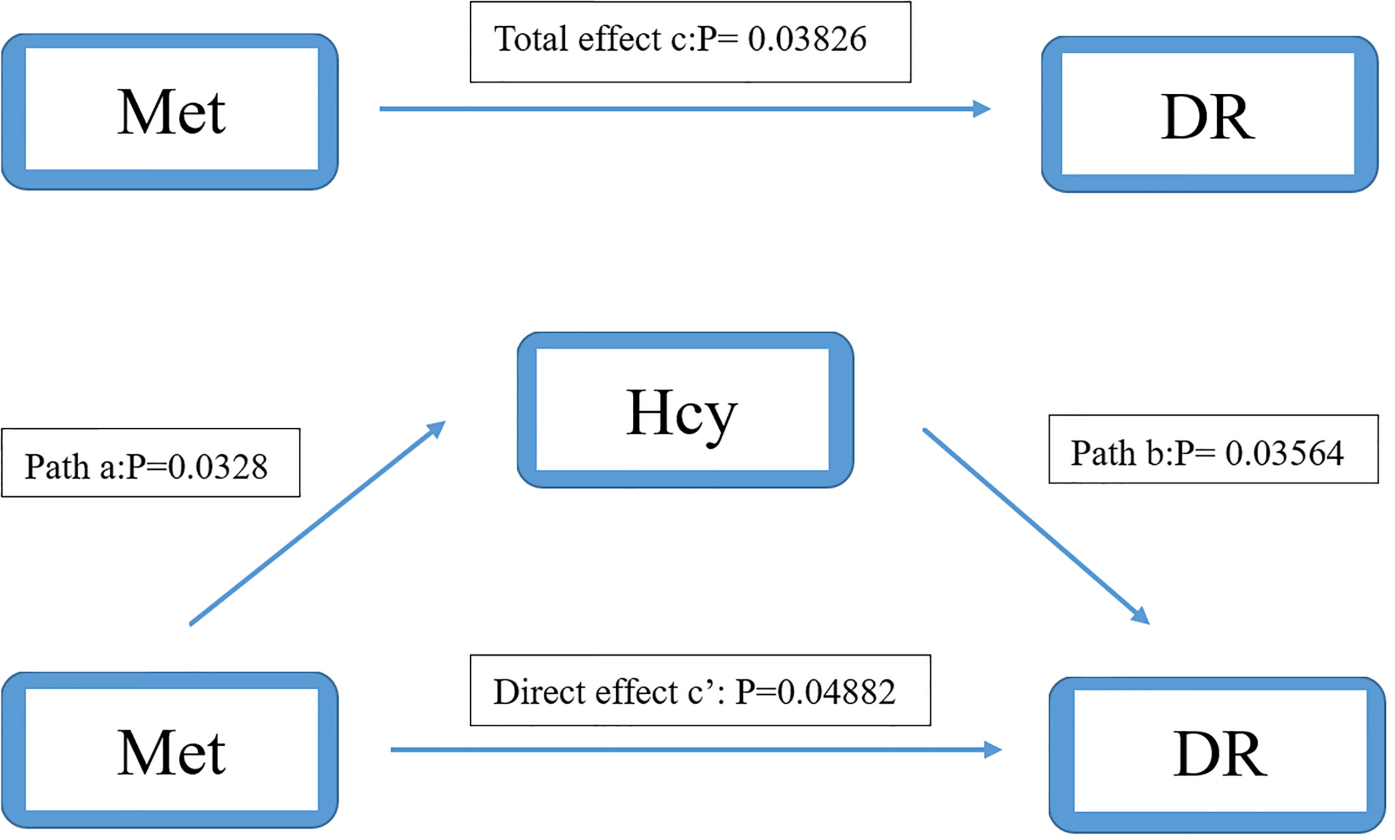
Mediation analysis of the relationship between Met and DR by Hcy. The mediation analysis adjusted for age, gender, body max index, systolic blood pressure, diastolic blood pressure, low-density lipoprotein cholesterol, high-density lipoprotein cholesterol, triglyceride, glycosylated hemoglobin, urinary creatinine and serum creatinine.

We used the causal steps approach (20) to analyze the mediating effect of the Hcy cycle pathway. **Tables 4**, **5** showed the results of the mediation analysis. In Model 1 (Hcy as mediator), the total effect of Met concentration on DR was significant (OR: 0.968, 95%CI: 0.938-0.998). The interaction between Met and Hcy was significant (OR: 1.047, 95%CI: 1.004-1.091). The effect of Hcy on DR was significant (OR: 0.964, 95%CI: 0.932-0.998). Meanwhile, the direct effect of Met on DR remained significant (OR: 0.969, 95%CI: 0.940-0.999) after adjusting for the Hcy (**Figure 4**). It shows that the mediation effect is significant in Model 1, and it is incomplete mediation.

**Table 4:** Mediation analysis of the relationship between Met and DR by Hcy.

	Met		
	Parameter estimate	OR (95%CI)	P
Total effect c	-0.0327	0.968 (0.938,0.998)	0.03826
Direct effect path c’	-0.0311	0.969 (0.940,0.999)	0.04882
Path a	0.0456	1.047 (1.004,1.091)	0.032820
Path b	-0.0367	0.964 (0.932,0.998)	0.03564

Adjusted for age, gender, body mass index., systolic blood pressure, diastolic blood pressure, low-density lipoprotein cholesterol, high-density lipoprotein cholesterol, triglyceride, total cholesterol, glycosylated hemoglobin, uric acid, serum creatinine.

Path c’ indicated the path from Met to DR (Outcome) when controlled for Hcy (Mediator).

Path a indicated the path from Met to Hcy (Mediator).

Path b indicated the path from Hcy (Mediator) to DR (Outcome).

**Table 5:** Mediation analysis of the relationship between Hcy and DR by Cys.

	Cys		
	Parameter estimate (95%CI)	OR (95%CI)	P
Total effect	-0.0367	0.964 (0.932,0.998)	0.03564
Direct effect path c’	-0.0349	0.966 (0.932,1.000)	0.05175
Path a	0.2543	1.290 (1.227,1.355)	< 0. 01
Path b	-0.0143	0.986 (0.956,1.017)	0.36798

Adjusted for age, gender, body mass index., systolic blood pressure, diastolic blood pressure, low-density lipoprotein cholesterol, high-density lipoprotein cholesterol, triglyceride, total cholesterol, glycosylated hemoglobin, uric acid, serum creatinine.

Path c’ indicated the path from Hcy to DR (Outcome) when controlled for Cys (Mediator).

Path a indicated the path from Hcy to Cys (Mediator).

Path b indicated the path from Cys (Mediator) to DR (Outcome).

**Figure 5:**
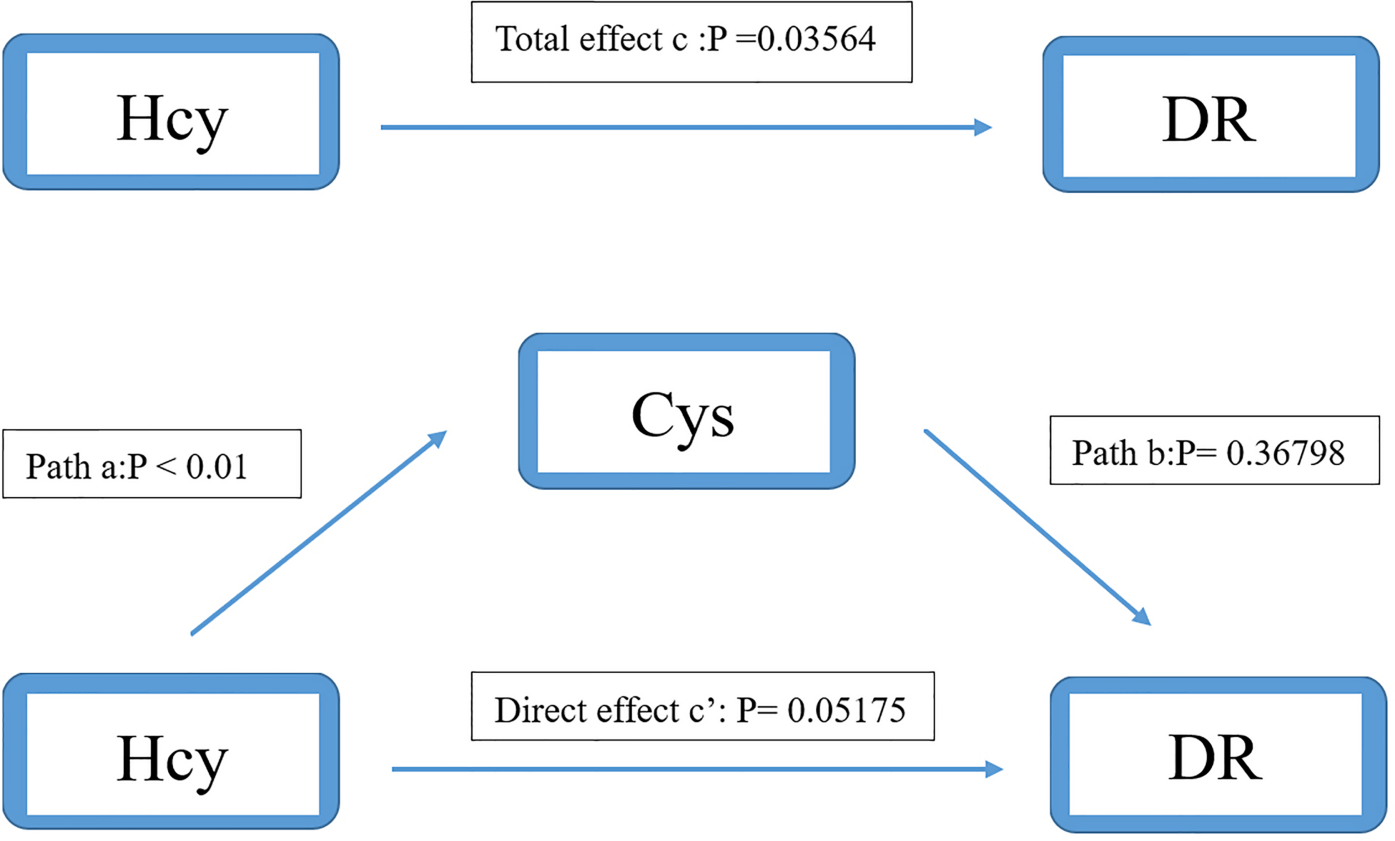
Mediation analysis of the relationship between Hcy and DR by Cys. The mediation analysis adjusted for age, gender, body max index, systolic blood pressure, diastolic blood pressure, low-density lipoprotein cholesterol, high-density lipoprotein cholesterol, triglyceride, glycosylated hemoglobin, urinary creatinine and serum creatinine.

In model 2 (Cys as mediator), the total effect of Hcy on DR was significant (OR: 0.964, 95%CI: 0.932-0.998). The interaction between Hcy and Cys was significant (OR: 1.290, 95%CI: 1.227-1.355). However, the effect of Cys on DR was not significant (OR: 0.986, 95%CI: 0.956-0.1.017). After adjusting for Cys, the direct effect of Hcy on DR was still not significant (OR: 0.966, 95%CI: 0.932-1.000) (**Figure 5**). It shows that there is no mediation effect in the pathway of model 2.

## Discussion

We divided the study population according to the slope change point of the RCS curve, and we obtained that the Hcy and DR are negatively correlated. In the study, it can be seen from the changing direction of the RCS curve that in people with low concentrations of Hcy, even if it is not reached the level of traditional high Hcy, there can still have an impact on the development of DR. And after the changing point of slope (7.7 μmol/L), the effect of Hcy on the risk of DR tends to be gentle, and it can be seen that the intervention before this point is more effective in reducing the risk of DR.

At present, many scholars have carried out relevant researches about the prediction of disease risk by Hcy level in the body. Hcy is considered to be related to a variety of diseases, especially the relationship with cardiovascular disease has been confirmed many times. In recent years, many scholars have begun to explore the effect of Hcy on diabetes and its complications, but the results of the relationship between Hcy and diabetes and its complications are controversial. Mainstream results suggest that Hcy is a risk factor for DR. A cross-sectional study in Hoorn identified Hcy as a risk factor for DR (21), and then they did a prospective cohort study which found that Hcy affects DR possibly by reducing transmethylation *in vivo (* 22). However, there are also some findings that are considered to be consistent with our results. A study found that Hcy above its threshold will increase the risk of DR (OR=1.66), while Hcy below the threshold become a protective factor for the DR (OR=0.83) (23). Although the result at low concentrations of Hcy was not statistically significant, this result was consistent with us in direction. We believe that the small sample sizes of the study (n=140) and the lack of adjustment for HDL-C, LDL-C, and UA may account for the no statistically significant results. Another study found that the median Hcy concentration in healthy people was 7.8 μmol/L, and the Hcy concentration in diabetic patients increased to 10.2 μmol/L. And the Hcy concentration continued to increase in patients with DR (24). Although this article does not conduct in-depth research on the effect of lower concentrations of Hcy on the risk of DR, it is basically consistent with the range of DR risk reduction in our study. It can be seen that the effect of Hcy on the DR presents a U-shaped curve. We believe that the possible reasons for the discrepancy in the study results are as follows: 1. different definitions of the normal range of Hcy in different studies (25). 2. The lower Hcy level in T2D patients than in healthy subjects in this study is a partial reflection of the U-shaped curve for the effect of Hcy on DR.

Hcy is an important intermediate product in the metabolism of Met to Cys. *In vivo*, a part of Hcy will regenerate Met in two ways: 1) Remethylation under the action of Met synthase and VB_12_ to generate Met and Tetrahydrogen folic acid (THFA). 2) is catalyzed by Betaine-homocysteine S-methyltransferase to generate Met and dimethylglycine. Another part forms cystathionine under the action of cystathionine β-synthase (CBS) and VB_6_ through the trans-sulfation pathway, and cystathionine forms Cys and α-ketobutyric acid under the action of γ-cystathionine lyase (26, 27). This is the main metabolic process of Hcy in the body. And the changes of Met and Cys *in vivo* also have influence on Hcy.

In the population grouped by Cys, low concentrations of Cys showed a significant additive interaction on the protective effect of Hcy on DR. It can be seen that low concentrations of Cys enhance the protective effect of Hcy [total population (OR: 0.83, 95%CI: 0.69-1), low Cys population (OR: 0.75, 95%CI: 0.61-0.94)]. Cys ​​is a sulfur-containing amino acid that supports the occurrence of various reactions in the body, such as the transformation and synthesis of proteins and glutathione (GSH) (28). GSH is a tripeptide containing γ-amide bond and sulfhydryl, which has antioxidant and detoxification effects and maintains normal immune function of the body. Cys ​​is indispensable in the synthesis of GSH. Insufficient Cys​​ can lead to decreased GSH levels in the body, which in turn reduces the body’s antioxidant capacity, leading to decreased immunity and aging of the body (29). It can be seen that the protective effect is stronger in the population with low Cys concentration, which means that the Hcy level is relatively higher in the normal range of our study. We believe that this additive effect on Hcy protection is temporary. Some studies have found that when the concentration of Hcy reaches about 10 μmol/L, the effect on the microvascular disease of the body becomes a negative effect (24). The conclusions obtained from our study are not in conflict with the existing results. In addition, the blood pressure of the case group was higher than that of the control group, and the difference between the systolic blood pressure was statistically significant. The result is consistent with previous studies showing that elevated blood pressure is a risk factor for diabetes and its complications (30, 31). Blood pressure control can reduce the risk of the disease.

In addition, the population grouped by Met concealed the heterogeneity of Hcy levels on the same pathway, making the relationship between Hcy and DR no longer significant. We think it is due to the mediating effect between Met, Hcy and DR pathway. We found a positive correlation between Met and Hcy concentration, indicating that the reduction of Met will synchronously lead to the decrease of Hcy concentration in individuals, and affect the difference of the real Hcy level in the actual population.

There are some published papers that are inconsistent with our results. We speculate that the heterogeneity of the population may had an impact on the differences in results. In addition, our study only reflects the relative changes of DR risk and does not represent the actual protection threshold. And due to the different definitions of population divisions, too low concentrations of Hcy may lead to changes in other metabolites in the body, resulting in inconsistent conclusions. In some published papers, most scholars use the definition of high Hcy (>15 μmol/L) to divide the population (32, 33) to study the effect of high Hcy on the human body. Few scholars have studied the relationship between the population with relatively low Hcy concentration and the pathogenesis of DR. And our research is based on this.

The accumulation of Hcy ​​in the body may be caused by two reasons: excessive production or abnormal metabolism of Hcy. Disorders of Met metabolizing enzymes and Hcy metabolizing enzymes (CBS, MS, MTHFR) can lead to disorders of Hcy metabolism, resulting in elevated Hcy concentrations. Similarly, vitamins B_2_, B_6_, B_12_ and folic acid are important coenzymes in the process of Hcy metabolism, and deficiency of these vitamins can lead to abnormal Hcy ​​metabolism, resulting in Hcy accumulation and causing HHcy (27). Studies have found that DR patients generally have lower concentrations of folic acid and VB_12_ (23), and folic acid is the main nutritional factor affecting Hcy concentration (34). Vitamin D deficiency is common in people with diabetes, and its deficiency increases the risk of microvascular disease (35). The retina is rich in polyunsaturated fatty acids and is very sensitive to oxidative stress. As an antioxidant, vitamin C can play a protective role in DR progression (36). In addition, studies have found that although the relationship between vitamin A and DR has shown inconsistent results in various countries, it has been demonstrated that V_A_ can significantly affect the development of DR (37). So, we believe that the protective phenomenon of Hcy in our study prompted other factors such as vitamin deficiency, drug use or disease state caused by abnormal Hcy metabolism. The use of metformin can reduce glycemia while leading to the deficiency of folic acid and VB_12_. Meanwhile, the metformin may affect Hcy metabolism, which in turn affects the progression of DR (10). Therefore, it can be seen from our research results that the progression of DR is not only the effect of one certain factor, but also the result of the joint action of many factors.

In addition, studies have found that when the population was binarized by Hcy concentration, the difference in the prevalence of DR between two groups was not statistically significant (25). This phenomenon is related to our study, which indirectly confirms that the risk of DR in the population with low Hcy concentration is not lower than that in the high Hcy population. It is suggested that we should consider the adverse effects of low Hcy on the levels of other substances related to the pathogenesis of DR in the human body.

Our research has important implications for clinical practice. At present, the clinical significance of high Hcy is mostly considered in clinical practice while little attention is paid to the joint effect of other factors reflected by Hcy. The study pointed out that the lower the level of Hcy in the body is not the better, and we should pay attention to the changes of other indicators reflected behind the low Hcy.

Our research also has shortcomings. First, due to the nature of the cross-sectional study, we could not prove the existence of a causal relationship, which requires more prospective cohort studies to confirm. Considering that there is still a lack of population studies on the Hcy pathway and the risk of DR, our study which as a cross-sectional study with a larger sample size, can provide certain etiological clues. DR caused by change in Hcy concentration or the change of Hcy concentration caused by DR *in vivo* have been a controversial topic in academic circles. Secondly, because of a lack of vitamin concentration and drug use, it is impossible to verify the effect of vitamins on this cycle, and the effect of drugs on the relationship between Hcy and DR cannot be determined either. Furthermore, our cases did not distinguish the types of DR. The difference in Hcy concentrations between patients with non-proliferating diabetic retinopathy (NPDR) and proliferative diabetic retinopathy (PDR) has been shown to be significant in some studies (24), and more detailed and in-depth studies are needed on the concentration of Hcy in patients with different types of DR.

In conclusion, we found an inverse relationship between low concentrations of Hcy and the risk of DR in T2D patients, suggesting that the influence curve of Hcy on DR may be U-shaped. This relationship was mainly influenced by the interaction of Cys *in vivo* and by changes in Met concentration. It may be a reflection of the lack of indicators such as vitamins in the body. Further experimental studies are needed to determine the role of Hcy in the pathogenesis of DR.

## Data Availability Statement

The raw data supporting the conclusions of this article will be made available by the authors, without undue reservation.

## Ethics Statement

Ethical review and approval was not required for the study on human participants in accordance with the local legislation and institutional requirements. The patients/participants provided their written informed consent to participate in this study. Written informed consent was obtained from the individual(s) for the publication of any potentially identifiable images or data included in this article.

## Author Contributions

W-ML is mainly responsible for writing the article, Z-ZF provides data support, and all the members are responsible for data analysis and collection. All authors contributed to the article and approved the submitted version.

## Funding

This work was supported by the national key research and development program of China (2021YFA1301200, 2021YFA1301202), Special Fund of State Key Joint Laboratory of Environment Simulation and Pollution Control. The funders had no role in study design, data collection and analysis, decision to publish, or preparation of the manuscript.

## Conflict of Interest

The authors declare that the research was conducted in the absence of any commercial or financial relationships that could be construed as a potential conflict of interest.

## Publisher’s Note

All claims expressed in this article are solely those of the authors and do not necessarily represent those of their affiliated organizations, or those of the publisher, the editors and the reviewers. Any product that may be evaluated in this article, or claim that may be made by its manufacturer, is not guaranteed or endorsed by the publisher.

## References

[1] International Diabetes Federation. (2021) E. coli. Available at: https://www.diabetesatlas.org [Accessed May 22, 2022].

[2] Ma. Epidemiology of Diabetes and Diabetic Complications in China. Diabetologia (2018) 61:1249–60. doi: 10.1007/s00125-018-4557-7 29392352

[3] Hou Cai Jia Shi. Risk Factors and Prevalence of Diabetic Retinopathy: A Protocol for Meta-Analysis. Med (Baltimore) (2020) 99:e22695. doi: 10.1097/MD.0000000000022695 PMC7571993 33080719

[4] Flaxman Bourne Resnikoff Ackland Braithwaite Cicinelli. Taylor and Vision Loss Expert Group of the Global Burden of Disease. Global Causes of Blindness and Distance Vision Impairment 1990-2020: A Systematic Review and Meta-Analysis. Lancet Glob Health (2017) 5:e1221–34. doi: 10.1016/S2214-109X(17)30393-5 29032195

[5] Yin Zhang Ren Su Sun. Prevalence and Risk Factors of Diabetic Retinopathy in Diabetic Patients: A Community Based Cross-Sectional Study. Med (Baltimore) (2020) 99:e19236. doi: 10.1097/MD.0000000000019236 PMC7478682 32118727

[6] Chen Gardner. A Critical Review: Psychophysical Assessments of Diabetic Retinopathy. Surv Ophthalmol (2021) 66:213–30. doi: 10.1016/j.survophthal.2020.08.003 PMC7914308 32866468

[7] Satyanarayana Balakrishna Pitla Reddy Mudili Lopamudra. Status of B-Vitamins and Homocysteine in Diabetic Retinopathy: Association With Vitamin-B12 Deficiency and Hyperhomocysteinemia. PloS One (2011) 6:e26747. doi: 10.1371/journal.pone.0026747 22069468 PMC3206053

[8] Wang Du Wang Zhang. Therapeutic Investigation of Quercetin Nanomedicine in a Zebrafish Model of Diabetic Retinopathy. BioMed Pharmacother (2020) 130:110573. doi: 10.1016/j.biopha.2020.110573 32745912

[9] Simo-Servat Hernandez Simo. Diabetic Retinopathy in the Context of Patients With Diabetes. Ophthalmic Res (2019) 62:211–7. doi: 10.1159/000499541 31129667

[10] Malaguarnera Gagliano Salomone Giordano Bucolo Pappalardo. Folate Status in Type 2 Diabetic Patients With and Without Retinopathy. Clin Ophthalmol (2015) 9:1437–42. doi: 10.2147/OPTH.S77538 PMC4536839 26300625

[11] Simo-Servat Simo Hernandez. Circulating Biomarkers of Diabetic Retinopathy: An Overview Based on Physiopathology. J Diabetes Res (2016) 2016:5263798. doi: 10.1155/2016/5263798 27376090 PMC4916280

[12] Keller Klawitter Hildreth Christians Putnam Kohrt. Elevated Plasma Homocysteine and Cysteine are Associated With Endothelial Dysfunction Across Menopausal Stages in Healthy Women. J Appl Physiol 1985 (2019) 126:1533–40. doi: 10.1152/japplphysiol.00819.2018 PMC6620665 30896357

[13] He Zeng Li Feng Liu Liu. Homocysteine Impairs Coronary Artery Endothelial Function by Inhibiting Tetrahydrobiopterin in Patients With Hyperhomocysteinemia. Am J Physiol Endocrinol Metab (2010) 299:E1061–5. doi: 10.1152/ajpendo.00367.2010 20858749

[14] Reed Nijhout Sparks Ulrich. A Mathematical Model of the Methionine Cycle. J Theor Biol (2004) 226:33–43. doi: 10.1016/j.jtbi.2003.08.001 14637052

[15] Xu Wu Liu Liu Wang Yu. Relationship Between Homocysteine Level and Diabetic Retinopathy: A Systematic Review and Meta-Analysis. Diagn Pathol (2014) 9:167. doi: 10.1186/s13000-014-0167-y 25257241 PMC4207897

[16] Tawfik Mohamed Elsherbiny DeAngelis Bartoli Al-Shabrawey. Homocysteine: A Potential Biomarker for Diabetic Retinopathy. J Clin Med (2019) 8:121. doi: 10.3390/jcm8010121 PMC6352029 30669482

[17] Alberti Zimmet. Definition, Diagnosis and Classification of Diabetes Mellitus and its Complications. Part 1: Diagnosis and Classification of Diabetes Mellitus Provisional Report of a WHO Consultation. Diabetes Med (1998) 15:539–53. doi: 10.1002/(SICI)1096-9136(199807)15:7<539::AID-DIA668>3.0.CO;2-S 9686693

[18] Faselis Katsimardou Imprialos Deligkaris Kallistratos Dimitriadis. Microvascular Complications of Type 2 Diabetes Mellitus. Curr Vasc Pharmacol (2020) 18:117–24. doi: 10.2174/1570161117666190502103733 31057114

[19] Chen Lu Department of Disease Control Ministry of Health. The Guidelines for Prevention and Control of Overweight and Obesity in Chinese Adults. BioMed Environ Sci (2004) 17:1–36. 15807475

[20] MacKinnon Fairchild Fritz. Mediation Analysis. Annu Rev Psychol (2007) 58:593–614. doi: 10.1146/annurev.psych.58.110405.085542 16968208 PMC2819368

[21] Hoogeveen Kostense Eysink Polak Beks Jakobs. Hyperhomocysteinemia is Associated With the Presence of Retinopathy in Type 2 Diabetes Mellitus: The Hoorn Study. Arch Intern Med (2000) 160:2984–90. doi: 10.1001/archinte.160.19.2984 11041907

[22] Van Hecke Dekker Nijpels Teerlink Jakobs Stolk. Homocysteine, S-Adenosylmethionine and S-Adenosylhomocysteine are Associated With Retinal Microvascular Abnormalities: The Hoorn Study. Clin Sci (Lond) (2008) 114:479–87. doi: 10.1042/CS20070275 17956228

[23] Fotiou Raptis Apergis Dimitriadis Vergados Theodossiadis. Vitamin Status as a Determinant of Serum Homocysteine Concentration in Type 2 Diabetic Retinopathy. J Diabetes Res (2014) 2014:807209. doi: 10.1155/2014/807209 25006590 PMC4071945

[24] Malaguarnera Gagliano Giordano Salomone Vacante Bucolo. Homocysteine Serum Levels in Diabetic Patients With non Proliferative, Proliferative and Without Retinopathy. BioMed Res Int (2014) 2014:191497. doi: 10.1155/2014/191497 24877066 PMC4022262

[25] Gupta John Rebekah John. Role of Hyperhomocysteinemia in Proliferative Diabetic Retinopathy: A Case-Control Study. Indian J Ophthalmol (2018) 66:1435–40. doi: 10.4103/ijo.IJO_350_18 PMC6173030 30249828

[26] Kumar Palfrey Pathak Kadowitz Gettys Murthy. The Metabolism and Significance of Homocysteine in Nutrition and Health. Nutr Metab (Lond) (2017) 14:78. doi: 10.1186/s12986-017-0233-z 29299040 PMC5741875

[27] Kim Kim Roh Kwon. Causes of Hyperhomocysteinemia and its Pathological Significance. Arch Pharm Res (2018) 41:372–83. doi: 10.1007/s12272-018-1016-4 29552692

[28] Stipanuk Dominy Lee Coloso. Mammalian Cysteine Metabolism: New Insights Into Regulation of Cysteine Metabolism. J Nutr (2006) 136:1652S–9S. doi: 10.1093/jn/136.6.1652S 16702335

[29] Forman Zhang Rinna. Glutathione: Overview of its Protective Roles, Measurement, and Biosynthesis. Mol Aspects Med (2009) 30:1–12. doi: 10.1016/j.mam.2008.08.006 18796312 PMC2696075

[30] Tight Blood Pressure Control and Risk of Macrovascular and Microvascular Complications in Type 2 Diabetes: UKPDS 38. UK Prospective Diabetes Study Group. BMJ (1998) 317:703–13. PMC28659 9732337

[31] Wang Cui Xu Xu. Association Between Plasma Homocysteine and Progression of Early Nephropathy in Type 2 Diabetic Patients. Int J Clin (2015) 8:11174–80. PMC4565303 26379920

[32] Tawfik Samra Elsherbiny Al-Shabrawey. Implication of Hyperhomocysteinemia in Blood Retinal Barrier (BRB) Dysfunction. Biomolecules (2020) 10:1119. doi: 10.3390/biom10081119 PMC7463551 32751132

[33] Abdella Mojiminiyi Akanji Moussa. Associations of Plasma Homocysteine Concentration in Subjects With Type 2 Diabetes Mellitus. Acta Diabetol (2002), 183–90. doi: 10.1007/s005920200033 12486492

[34] Abuawad Bozack Saxena Gamble. Nutrition, One-Carbon Metabolism and Arsenic Methylation. Toxicology (2021), 152803. doi: 10.1016/j.tox.2021.152803 33905762 PMC8349595

[35] Tecilazich Formenti Giustina. Role of Vitamin D in Diabetic Retinopathy: Pathophysiological and Clinical Aspects. Rev Endocr Metab Disord (2021), 715–27. doi: 10.1007/s11154-020-09575-4 PMC7538371 33026598

[36] Lopes de Jesus Atallah Valente Moca Trevisani. Vitamin C and Superoxide Dismutase (SOD) for Diabetic Retinopathy. Cochrane Database Syst Rev (2008), CD006695. doi: 10.1002/14651858.CD006695.pub2 18254110

[37] Zhang Wang Hu Chen. Vitamin A and Diabetes. J Med Food (2021) 24(8), 775–85. doi: 10.1089/jmf.2020.0147 33232625

