# Supplementary material for: The Association of Homocysteine and Diabetic Retinopathy in Homocysteine Cycle in Chinese Patients With Type 2 Diabetes
Source: Front Endocrinol (Lausanne). 2022 Jun 29;13:883845. doi: 10.3389/fendo.2022.883845 (PMC9276920; doi:10.3389/fendo.2022.883845)
Supplement: Supplementary file 1 [file DataSheet_1.pdf]

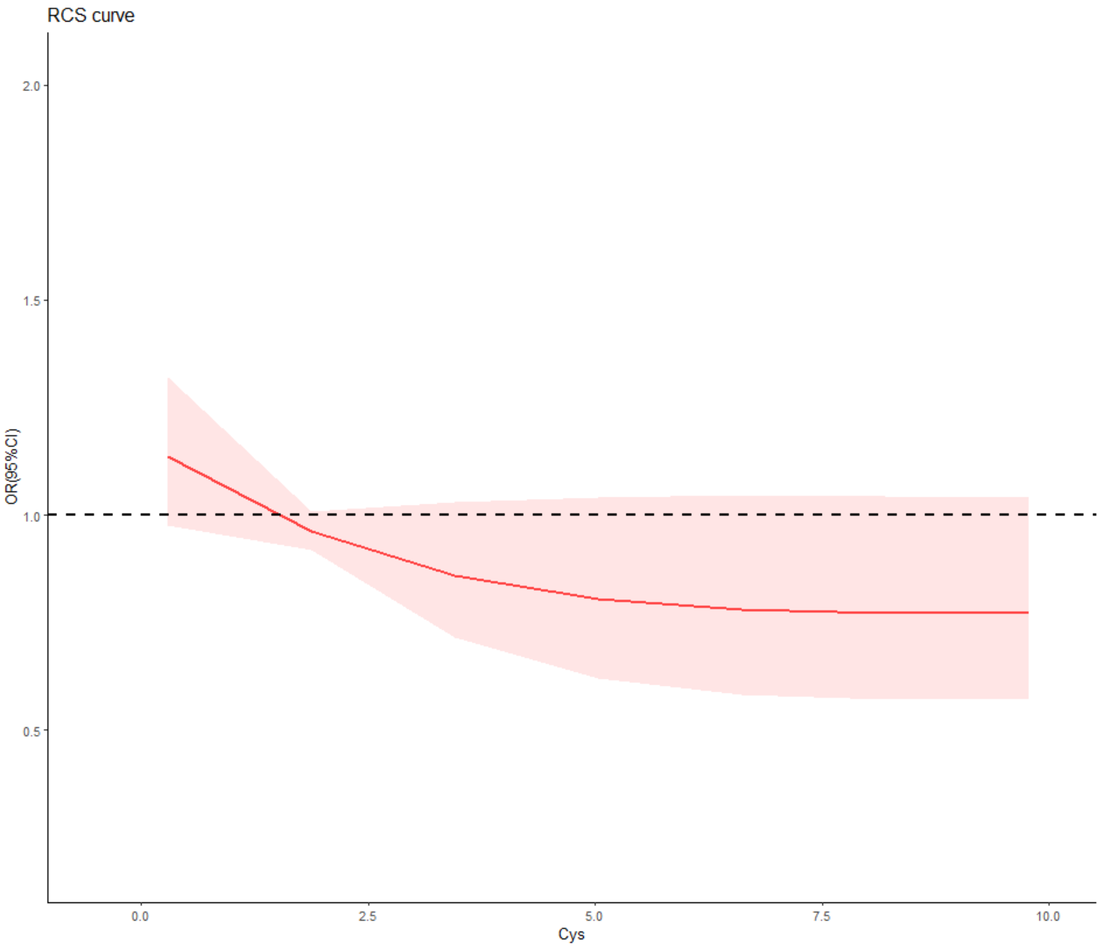

Figure S1 | The relationship between Cys concentration and DR risk in T2D patients. The red curve was derived from multivariate analysis that adjusted for age, gender, body mass index, systolic blood pressure, diastolic blood pressure, low-density lipoprotein cholesterol, high-density lipoprotein cholesterol, triglyceride, glycosylated hemoglobin, urinary creatinine and serum creatinine.

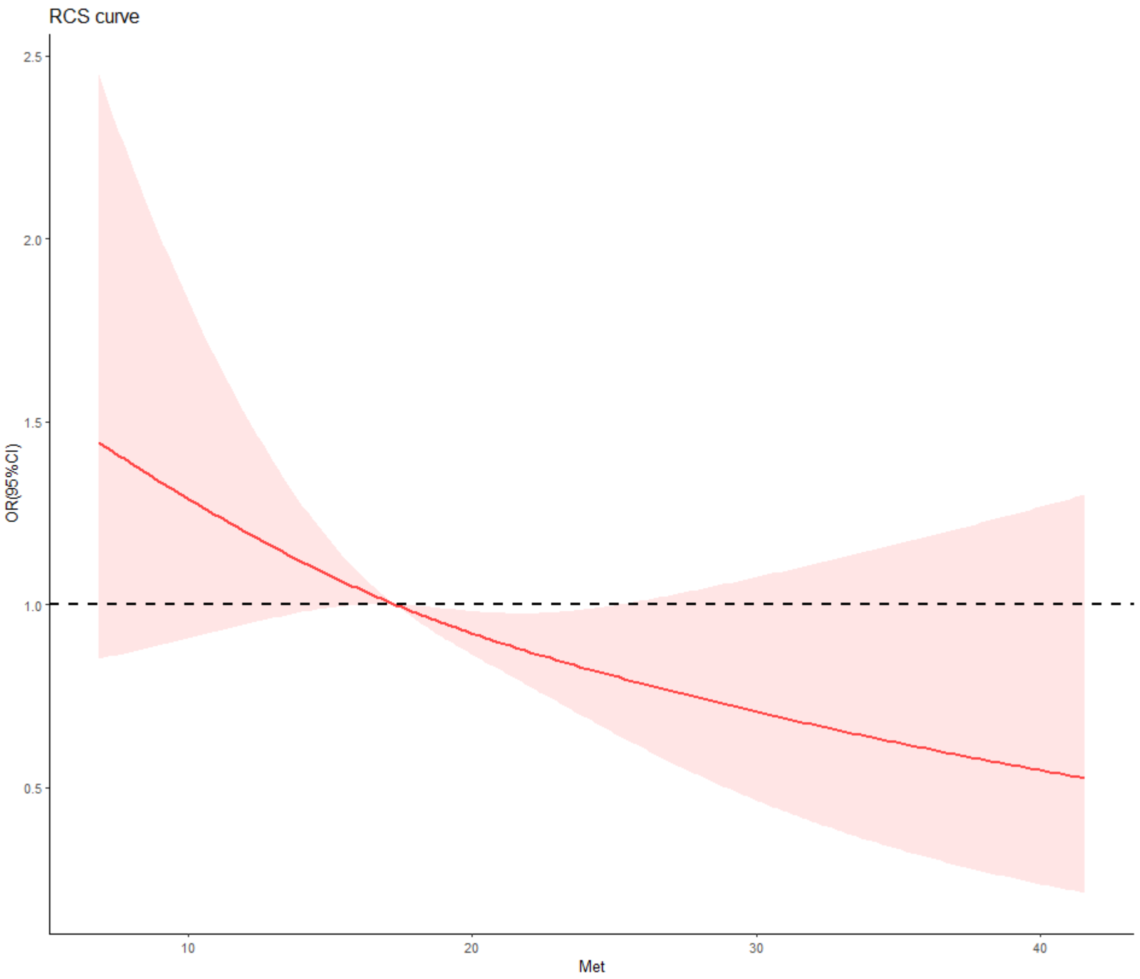

Figure S1 | The relationship between Met concentration and DR risk in T2D patients. The red curve was derived from multivariate analysis that adjusted for age, gender, body mass index, systolic blood pressure, diastolic blood pressure, low-density lipoprotein cholesterol, high-density lipoprotein cholesterol, triglyceride, glycosylated hemoglobin, urinary creatinine and serum creatinine.
